# Precision cancer mouse models through genome editing with CRISPR-Cas9

**DOI:** 10.1186/s13073-015-0178-7

**Published:** 2015-06-09

**Authors:** Haiwei Mou, Zachary Kennedy, Daniel G. Anderson, Hao Yin, Wen Xue

**Affiliations:** RNA Therapeutics Institute and Program in Molecular Medicine, University of Massachusetts Medical School, Worcester, MA 01605 USA; David H. Koch Institute for Integrative Cancer Research, Massachusetts Institute of Technology, Cambridge, MA 02142 USA; Department of Chemical Engineering, Massachusetts Institute of Technology, Cambridge, MA 02142 USA; Harvard-MIT Division of Health Sciences & Technology, Cambridge, MA 02139 USA; Institute of Medical Engineering and Science, Massachusetts Institute of Technology, Cambridge, MA 02142 USA

## Abstract

The cancer genome is highly complex, with hundreds of point mutations, translocations, and chromosome gains and losses per tumor. To understand the effects of these alterations, precise models are needed. Traditional approaches to the construction of mouse models are time-consuming and laborious, requiring manipulation of embryonic stem cells and multiple steps. The recent development of the clustered regularly interspersed short palindromic repeats (CRISPR)-Cas9 system, a powerful genome-editing tool for efficient and precise genome engineering in cultured mammalian cells and animals, is transforming mouse-model generation. Here, we review how CRISPR-Cas9 has been used to create germline and somatic mouse models with point mutations, deletions and complex chromosomal rearrangements. We highlight the progress and challenges of such approaches, and how these models can be used to understand the evolution and progression of individual tumors and identify new strategies for cancer treatment. The generation of precision cancer mouse models through genome editing will provide a rapid avenue for functional cancer genomics and pave the way for precision cancer medicine.

## The need for precision cancer models

The complexity of the cancer genome [1, 2] vastly exceeds the textbook view of a homogeneous mass of cells with a handful of genetic mutations. A recent genomic study using 4724 tumor-normal pairs across 21 cancer types showed that each tumor-normal pair has, on average, 672 point mutations, translocations and numerous chromosome gains and losses [3]. To identify oncogenes and tumor suppressor genes, precise modification of genomic DNA will be needed to produce mutations that can be carefully examined [1, 2, 4]. This will be particularly important for the validation of the large numbers of candidate cancer genes identified by The Cancer Genome Atlas (TCGA) at the US National Institutes of Health (NIH). TCGA aims to comprehensively characterize the mutational landscape and genomic features of cancer. Because a subset of mutations in cancer are not relevant to cancer progression (so-called ‘passenger genes’), it is important to functionally validate candidate genes to identify those that are relevant to cancer progression (‘cancer driver genes’). In the face of such a complex genomic landscape, there is a need for simple and flexible genetic methods to generate mouse models that can identify functional cancer driver genes among the vast number of passenger mutations.

Traditional cancer mouse models mostly rely on genetically engineered transgenes or homologous recombination in embryonic stem (ES) cells [5]. By injecting genetically modified ES cells into wild-type blastocysts, chimeric animals with altered germlines are generated; this is a costly and time-consuming way to produce single-gene knockout mice or double-mutant mice. Moreover, for most other mammalian species, there are no established ES cell lines, which limits the studies that can be undertaken in many species [5, 6]. As the field moved towards more precise genome editing, programmable nucleases, including zinc-finger nucleases (ZFNs) and transcription-activator-like effector nucleases (TALENs) [7], have been developed. These approaches have had some success; for example, a ZFN-based approach for the HIV co-receptor gene *CCR5* is in clinical trials. However, both ZFNs and TALENs are nuclease-based designs that are difficult to construct, and the efficiency of targeting varies substantially, making these laborious approaches.

**Fig. 1:**
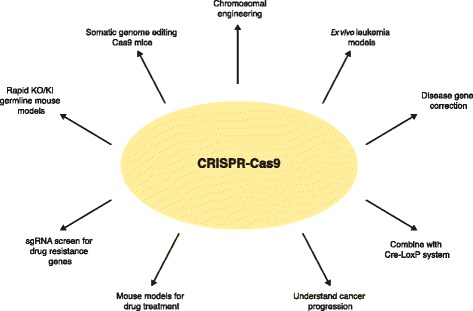
Applications of CRISPR/genome editing for precision cancer models. CRISPR has been used to generate genetically modified mouse models such as KO/KI germline models, somatic genome editing models and mouse models for drug treatment. CRISPR has been proved a useful tool to investigate chromosomal engineering, generate ex vivo leukemia models and identify drug resistance genes through genome editing of cell lines. CRISPR has also been used to correct disease-associated genes through homology-directed repair pathway. In combination with traditional Cre-*LoxP* system, CRISPR can generate conditional KO/KI mouse models and further the understanding of cancer progression. KO, knockout; KI, knock-in; sgRNA, single guide RNA

**Fig. 2:**
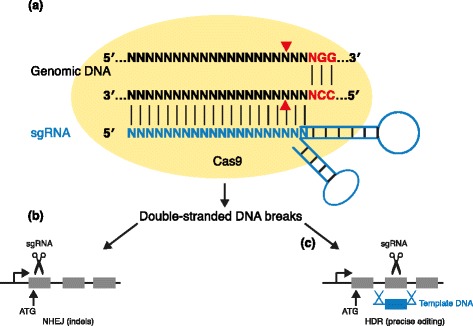
CRISPR-Cas9 mechanism. **a** A single guide RNA (sgRNA) is a fusion between crRNA (CRISPR RNA) and tracrRNA (trans-activating CRISPR RNA). This complex recognizes the protospacer-adjacent motif (PAM) sequence and the complementary 20-nucleotide upstream genomic sequence. Cas9 cuts approximately three nucleotides upstream of the PAM to induce DNA double-strand breaks. Then, the cellular DNA repair system, either non-homologous end joining (NHEJ) or homology-directed repair (HDR), results in indels or precise editing, respectively. Cas9 proteins from different bacteria recognize different PAM sequences; *S. pyogenes* Cas9 recognizes ‘NGG’ PAM and the weaker ‘NAG’ PAM. **b**, **c** Key underlying principles for CRISPR-Cas approaches. **b** sgRNA targeting tumor suppressor genes can lead to loss-of-function frameshift mutations through NHEJ. **c** Template DNA can be used to introduce precise genome editing through HDR (for example, oncogene mutations). Dashed lines denote homologous recombination

Recently, the CRISPR-Cas9 system has transformed genome editing (Fig. 1). CRISPR-Cas9 is an RNA-guided nuclease involved in adaptive immunity in bacteria and archaea [4, 8–10] (Fig. 2a and Box 1). Cas9 is guided by programmable RNA known as the single guide RNA (sgRNA) [11–15]. The Cas9/sgRNA complex recognizes the complementary 20-nucleotide genomic sequence with a downstream protospacer-adjacent motif (PAM) sequence (Fig. 2a). Cas9 proteins from different bacteria recognize different PAM sequences; for example, *Streptococcus pyogenes* Cas9 recognizes the ‘NGG’ PAM and the weaker ‘NAG’ PAM sequences [16]. Because most studies use *S. pyogenes* Cas9 as a genome-editing tool, we will use ‘CRISPR-Cas9’ to represent this Cas9 species. Cas9 cuts approximately three nucleotides upstream of the PAM sequence to induce double-strand DNA breaks (DSBs). These breaks are then repaired by the cells’ DNA damage repair mechanisms using either the error-prone non-homologous end-joining (NHEJ) pathway, which gives rise to small insertions and deletions (indels) (Fig. 2b), or the homology-directed repair (HDR) pathway that, in the presence of a donor double-stranded or single-stranded DNA, can lead to precise DNA modification (Fig. 2c).

CRISPR-Cas9 genome editing has made it easy, fast and effective to build precision cancer models. It has already been used to generate knockout or knock-in mouse models in ES cells and zygotes [17, 18]. *In vivo* delivery of CRISPR has been used to correct disease genes in mouse liver [19], and the CRISPR technique has been used to engineer structural variations, such as translocations, deletions and inversions [20–23]. It has also shown promise as a tool for modeling cooperative genetic events in lung cancer [24, 25], performing genome-scale screening [24, 25], and studying transcription regulation [26, 27] via catalytically deactivated Cas9 (dCas9). CRISPR-Cas9 is a rapid and flexible system that has the potential to speed up building of precision mouse models.

Here, we summarize the recent innovations that have permitted multiple applications of the CRISPR genome-editing system for building precision cancer mouse models and their implications for advancing cancer biology (Table 1). We will highlight a broad range of cancer-specific approaches to CRISPR gene-editing and discuss the efficacy of such approaches for generating cancer mouse models and how they can be used to further the understanding of tumor biology.

**Table 1:** List of CRISPR-generated animal models useful for the study of oncology and disease

	Animal	Vehicle for delivery	Target tissue	Delivery	Genes targeted	Utility	Reference
Germline	Mouse	mRNA, sgRNA and donor DNA	Embryo	One-cell embryo injection	*Nanog*, *Sox2*, *Oct4*	Generation of reporter genes	[18]
	Mouse	mRNA, sgRNA and donor DNA	Embryo	One-cell embryo injection	*Mecp2* Cre-LoxP conditional mutant	Rett syndrome	[18]
	Mouse	mRNA and sgRNA	Embryo	One-cell embryo injection	*Tet1* and *Tet2* and others	Epistatic gene interactions	[17]
	Rat	mRNA and sgRNA	Embryo	One-cell embryo injection	*	**	[29]
	Cynomolgus monkey	mRNA and sgRNA	Embryo	One-cell embryo injection	*Pparg* and *Rag1*	**	[30]
	Mouse	Plasmid DNA	ES cell	Dox-inducible Cas9 alleles	*p53*, *Apc*, *Pten*	Colon cancer	[36]
Somatic	Mouse	Plasmid DNA	Liver	Hydrodynamic injection	*Pten*, *p53* and β-catenin	Hepatocellular cancer	[25]
	Mouse	Adenovirus	Liver	Intravenous injection	*Pcsk9*	Cardiovascular disease	[32]
	Mouse	Adenovirus	Liver	Intravenous injection	*Cebpa*	Liver function	[33]
	Mouse	AAV	Brain	Stereotactic delivery	*Mecp2*	Rett syndrome	[34]
	Mouse	AAV	Brain	Stereotactic delivery	*Dnmt1*, *Dnmt3a* and *Dnmt3b*	Learning/memory	[34]
	Mouse	AAV	Brain	Stereotactic injection	*NeuN* (*Rbfox3*)	**	[35]
	Mouse	sgRNA in nanoparticle	Pulmonary/cardiovascular	Intravenous injection	*Icam2*	**	[35]
	Mouse	AAV, AAV donor template	Lung	Intranasal/intratrachael delivery	*p53* and *Lkb1*, *Kras*	Non-small-cell lung cancer	[35]
	Mouse	Lentivirus	Lung	Intratracheal delivery	*Nkx2.1*, *Pten*, *Apc*	Non-small-cell lung cancer	[24]
	Mouse	Adenovirus Lentivirus	Lung	Intratracheal delivery	*Eml4-Alk*	Non-small cell lung cancer	[22, 39]
Lymphoma	Mouse	Lentivirus	HSPCs ex vivo	Intravenous injection of Cas9-edited human HSPCs	*TET2*, *DNMT3A*, *RUNX1*, *NF1*, *EZH2*	AML	[41]
	Mouse	DNA electroporation	Fetal-liver HSCs ex vivo	Intravenous injection of Cas9-edited HSCs	*Mll3*	AML	[43]
	Mouse	Lentivirus	Fetal-liver HSCs ex vivo	Intravenous injection of Cas9-edited HSCs	*Mcl-1*, *p53*	Dox-inducible Burkitt lymphoma model	[44]
	Mouse	Retrovirus	Lymphoma cells ex vivo	Intravenous injection of Cas9-edited mouse *Arf* ^*−/−*^ *; Eμ-myc* cells	*p53*	Role of p53 in chemotherapy resistance	[42]

*Paper reports on protocol for rat-specific editing, does not investigate targeting of specific genes. **Applications of model for study of disease not critical focus of experiment. Donor DNA used as template for homologous recombination. AAV, adeno-associated virus; AML, acute myelogenous leukemia; Dox, doxycycline; ES cell, embryonic stem cell; HSPC, hematopoietic stem and progenitor cell; HSC, hematopoietic stem cell; sgRNA, single guide RNA

## Generating mouse models using the CRISPR system

The CRISPR system can be used to induce genetic mutations in as little as 4 weeks [26] and provides a flexible platform for functional annotation of the cancer genome. The general principles of applying the CRISPR-Cas9 system are as follows. First, an sgRNA harboring a 20-nucleotide genomic sequence from a gene of interest needs to be designed, and second, NHEJ or HDR is used to introduce indels or precise repair, respectively (Fig. 2, Box 1). Of note, HDR efficiency is usually low and several studies have demonstrated methods to increase HDR efficiency by inhibiting NHEJ enzymes [27]. The system can be customized for specific projects, mainly through design of the sgRNA strand. For example, an sgRNA that targets a protein-coding region can produce loss-of-function frameshift indel mutations through NHEJ (Fig. 2b) and sgRNA/template DNA can introduce precise genome editing through HDR (Fig. 2c). Currently, two complementary approaches, germline and somatic, are being used to build mouse models via genome editing.

### Germline CRISPR mouse models

**Fig. 3:**
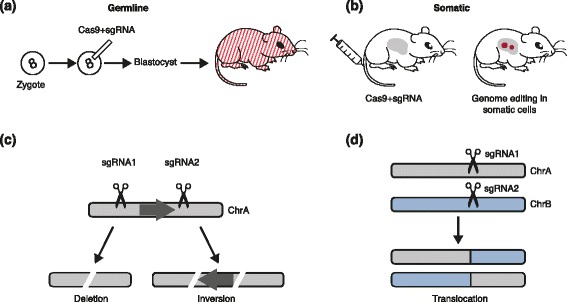
Rapid generation of cancer models in mouse through genome editing. **a** Germline CRISPR mouse models. Cas9 and single guide RNA (sgRNA) can be microinjected into mouse zygotes. The resulting mouse will carry cells harboring CRISPR-mediated indels or homology-directed repair. This method can generate mosaic mice. **b** Somatic CRISPR mouse models. Cas9 and sgRNA can be delivered to mouse tissue in vivo, for example through hydrodynamic injection to the liver or viral vehicles to various tissue. **c** Two sgRNAs targeting one chromosome can lead to deletion or inversion between sgRNA cutting sites. **d** Two sgRNAs targeting two chromosomes can lead to chromosomal translocation, allowing rapid modeling of cancer-associated chromosomal rearrangement. Chr, chromosome

Germline mouse models are developed by introducing cancer mutations into mouse ES cells or developing embryos. This method can generate germline-transmittable genetic alleles, allowing maintenance and breeding of the established alleles through mouse husbandry. CRISPR-Cas9 can precisely introduce DSBs in a one-cell-stage embryo, accelerating the generation of genetically modified mice as this stage is no longer dependent on the generation of suitable ES cells [26, 28]. For example, mice carrying a tag or a fluorescent reporter construct in *Nanog*, *Sox2* and *Oct4* (important stem cell genes) as well as a *Mecp2* conditional mutation (a Rett syndrome gene) were generated by one-step co-injection of zygotes with *Cas9* mRNA, different sgRNAs and DNA vectors (Fig. 3a) [18].

Moreover, CRISPR germline editing has been used to generate mice with biallelic mutations in the epigenetic genes*Tet1* and *Tet2* by one-step co-injection of *Cas9* mRNA and sgRNAs [15]. It has also been used to simultaneously disrupt five genes (*Tet1*, *2*, *3* and *Sry*, *Uty-8* alleles) in mouse ES cells [17]. Furthermore, CRISPR was used to generate precise point mutations in two targeted genes simultaneously in the presence of single-stranded DNA oligonucleotides with point mutations as templates for HDR repair. This is a useful approach for exploring redundant genes and epistatic gene interactions in vivo [17]. Of note, this one-step CRISPR injection system has also been used to precisely target genes in rat [29] and cynomolgus monkey [30] one-cell embryos.

### Somatic CRISPR mouse models

Most non-familial cancer is caused by the accumulation of genetic mutations in somatic cells. Somatic mouse cancer models recapitulate the somatic nature of cancer progression and bypass the embryonic lethality caused by whole-body knockout of certain genes. The large size of Cas9 (4 kilobases (kb)) is a challenge for delivery to somatic cells in mice. However, several plasmid delivery approaches (mainly to the liver because it is the most amenable organ to DNA delivery) and viral delivery approaches for CRISPR-Cas9 have been applied to generate somatic mouse models of cancer.

Hydrodynamic injection is a well-established method of delivering plasmids selectively to the liver in animal models. This method uses high-volume/high-pressure tail-vein injection to transiently express DNA in 20 to 30 % of mouse hepatocytes. For example, DNA plasmids encoding Cas9 and sgRNAs were delivered to the liver to target the tumor suppressor genes *Pten* and *p53*, alone or in combination (Fig. 3b) [25]. Strikingly, when *Pten* was mutated by CRISPR in hepatocytes, two liver phenotypes were observed: elevated Akt phosphorylation and lipid accumulation. Furthermore, simultaneous disruption of Pten and p53 induced tumor formation, phenocopying the effects of deletion of the genes using Cre-LoxP technology [25]. The feasibility of inducing point mutations in tumor suppressor genes and oncogenes in the liver was also demonstrated by co-injection of Cas9-sgRNA plasmids targeting β-catenin and a single-stranded DNA oligonucleotide donor carrying activating point mutations, resulting in hepatocytes with activation of β-catenin localizing in the nucleus [25]. Notably, a similar TALEN approach has been used to edit the β-catenin gene in mouse liver [31]. These advances in rapidly modeling liver cancer pave a new way for studies of functional cancer genomics.

In addition to plasmid-based delivery and TALEN-based approaches, various virus-based approaches have been developed to increase delivery efficiency and target organs other than the liver. For example, an adenovirus-based CRISPR-Cas9 expression system was used to introduce loss-of-function mutations in the endogenous *Pcsk9* gene, and resulted in decreased plasma Pcsk9 levels, increased hepatic low-density lipoprotein receptor levels and decreased plasma cholesterol levels, all of which are well known to be regulated by Pcsk9 [32]. Another group reported using adeno Cas9n (Cas9^D10A^ nickase capable of cutting only one DNA strand compared with wild-type Cas9, which can cut both strands, usually used to avoid off-target effects)-based delivery of CRISPR to mouse liver for disruption of *Cebpa*, a well-known metabolic transcription factor, and found a downstream molecule expression pattern similar to liver-specific *Cebpa* knockouts [33].

A Cas9/sgRNA delivery vector based on adeno-associated virus (AAV), with its non-pathogenic, potent and simple-to-engineer capability, has been used to target post-mitotic neurons in adult mice to elucidate the genetics of complex disorders that affect cognition and behavior. AAV vectors expressing Cas9 and sgRNA targeting a single gene (*Mecp2*, which causes the neurodevelopmental disorder Rett syndrome), as well as multiple genes (*Dnmt1*, *Dnmt3a* and *Dnmt3b* from the DNA methyltransferase family), in adult mouse brains have been stereotactically injected into the dentate gyrus, enabling reverse genetic studies of gene function in the brain [34]. This technique provides a potentially applicable method to model brain cancer by multiplex disruption of candidate genes.

A challenge in the field of CRISPR-based genome engineering is the delivery of the Cas9 endonuclease, which is large (4 kb). In an attempt to tackle this shortcoming, a Cre-dependent Cas9 knock-in mouse was generated and used for in vivo as well as *ex vivo* genome editing [35]. Using this system, a single AAV vector was delivered in the lung to generate loss-of-function mutations in *p53*, and *Lkb1* as well as HDR-mediated Kras^G12D^ mutations, which phenocopied the pathology of lung adenocarcinoma [35]. The Cas9 mouse provides a simplified model for somatic genome editing by expressing Cas9 in the germline, allowing constitutive and Cre-inducible Cas9 expression in organs of interest. Similarly, Lowe and colleagues [36] described the use of doxycycline-inducible Cas9 and Cas9^D10A^ mouse models to perform inducible genome editing in mice.

## Cancer-specific applications of CRISPR-Cas9

Recent cancer genome projects have identified a plethora of genomic alterations, such as deletions, inversions and translocations [3]. However, creating large structural variations using Cre-LoxP methods is time-consuming and therefore the function of many cancer-associated structural variations is not known. In addition, to fully explore cancer in a mouse model, it is necessary to look beyond solid tumors to leukemia models. In the past few months, several groups have demonstrated the potential of the CRISPR-Cas9 system for engineering large chromosomal changes and for *ex vivo* engineering.

### Engineering structural variations

Cell-line, ES-cell and somatic approaches have all been used to generate cancer- or disease-associated structural variations. For example, the engineering of CRISPR-mediated genomic deletions ranging from 1.3 kb to greater than 1 megabase (Mb) in mammalian cells has been reported and an inverse relationship exists between deletion frequency and deletion size [37]. Two sgRNAs that target one chromosome can lead to deletion or inversion between sgRNA cutting sites (Fig. 3c). Two sgRNAs targeting two chromosomes can lead to chromosomal translocation (Fig. 3d). These techniques allow rapid modeling of cancer-associated chromosomal rearrangement.

Chromosomal rearrangements, including *CD74-ROS1* translocation events and the *EML4-ALK* and *KIF5B-RET* inversion events, occur in lung cancer patients. These rearrangements have been generated by CRISPR-Cas9 technology [20, 38]. In addition, chromosomal translocations between the *EWSR1* and *FLI1* loci and between the *AML1* (also known as *RUNX1*) and *ETO* (also known as *RUNX1T1*) loci, which are involved in Ewing’s sarcoma and acute myeloid leukemia (AML), respectively, have been generated by CRISPR in human cell lines [23]. This provides an effective method to reproduce precise reciprocal tumor-associated chromosomal translocations to better understand the initiation of leukemia and sarcoma. Recently, Kraft and colleagues [21] reported that they had rearranged targeted genomic intervals from 1 kb to 1.6 Mb using the CRISPR-Cas9 system at six different loci associated with human disease, comprising *H2afy*, *Bmp2*, *Ihh*, *Pitx1*, *Laf4* (also known as *Aff3*) and *Epha4*. They observed deletions and inversions at all sites, and duplications in *Pitx1* and *Laf4* with 0.7 % and 28.1 % frequency, respectively. The deletion of 353 kb and 1.6 Mb genomic intervals at the *Laf4* and *Epha4* loci recapitulated human malformation syndromes and neurological phenotypes [21]. This study suggests that CRISPR can be used to genetically modify multiple genes from different loci and large genomic regions. The same strategy can be applied to mouse cancer models since cancer involves complex structural variants.

In addition to modeling chromosomal rearrangements in cell lines and ES cells, viral delivery of the CRISPR-Cas9 system to generate the *Eml4-Alk* rearrangement in non-small-cell lung cancers (NSCLCs) [22, 39] has been reported in mouse models. Our groups have recently shown that hydrodynamic injection of two sgRNAs led to deletion and inversion of a 50 kb *Pten* genomic region in mouse liver [40]. Together, these studies demonstrate that CRISPR is a feasible method to generate chromosomal rearrangements in vivo.

### Leukemia models

In addition to somatic genome engineering in vivo, several groups have reported efficient *ex vivo* engineering of hematopoietic stem cells (HSCs). HSCs are transduced with CRISPR-Cas9 viral vectors in vitro and transplanted into recipient mice, allowing for rapid generation of novel mouse models of hematopoietic malignancies [41–44]. For example, a recent report demonstrated efficient modification of up to five genes by delivering combinations of sgRNAs and Cas9 with a lentiviral vector in HSCs [41]. This led to clonal outgrowth and myeloid malignancy, thus generating models of AML. This study suggests that lentivirus-delivered sgRNA-Cas9 genome editing is another useful tool to engineer hematopoietic cancer models [41].

The above studies demonstrated that CRISPR can be used to engineer structural variants such as insertions, deletions, mutations and translocations, as well as to model leukemia. These advances pave the road to generating more complex cancer models.

## Generating complex models

Cancer involves a complex combination of mutations in tumor suppressor genes and oncogenes. Cancer genome sequencing studies have identified a variety of genetic alterations. However, not all the alterations contribute to tumorigenesis. Distinguishing cancer driver genes from passenger genes is a major challenge for cancer research.

Recent studies have demonstrated the power of combining the CRISPR-Cas9 system with well-studied conditional mouse models of cancer. Notably, a somatic genome engineering approach that combines Cre-dependent somatic activation of Kras^G12D^ [45–47] with CRISPR-Cas9-mediated genome editing of tumor suppressor genes has been shown to enable the rapid functional characterization of putative lung cancer genes using mouse models [24]. In this study, a pSECC lentiviral system was used to deliver both the CRISPR system and Cre recombinase, thus allowing for the examination of CRISPR-meditated mutations of genes in the context of well-studied Cre-LoxP lung cancer models. For instance, it was demonstrated that in *Kras*^LSL-G12D/+^ and *Kras*^LSL-G12D/+^; *p53*^fl/fl^ lung tumor models, loss of NK2 homeobox1 (*Nkx2-1*) [48] or *Pten* accelerated tumorigenesis [24]. Furthermore, this system has also been used to characterize the functional role of a novel lung tumor suppressor gene, adenomatous polyposis coli (*Apc*) [24]. Thus, combining CRISPR and Cre-LoxP models bypasses the laborious breeding of mouse alleles and significantly accelerates the pace of generation of complex mouse models.

The heterogeneity of tumor cells makes it difficult to trace which populations of cells eventually progress to malignancy and gain the ability to aggressively migrate and invade [49]. Because CRISPR induces indels at target genes in tumor cells [24, 25], we may be able to use these indels as ‘molecular barcodes’ to trace single-cell lineages and investigate the contribution of different populations of tumor cells to tumorigenesis and progression. For example, sgRNA targeting *Pten* has been shown to introduce diverse indels such as a ‘+C’ insertion and ‘-A’ and ‘-AA’ deletions at the sgRNA target site [25]. These indels could potentially serve as ‘DNA barcodes’ to differentiate tumor cell clones harboring unique CRISPR-induced indels.

## Precision models for cancer treatment and resistance

**Fig. 4:**
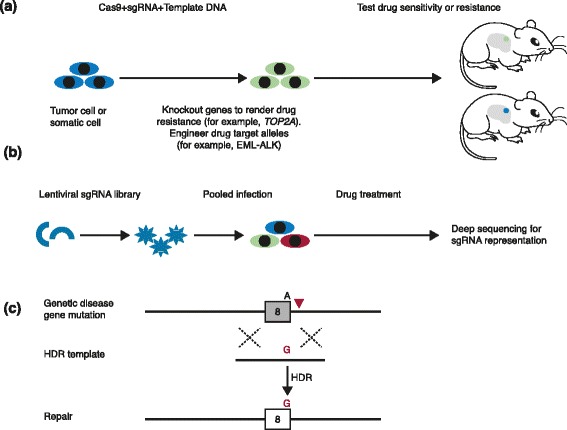
Precision models for cancer drug treatment and resistance. **a** CRISPR-induced gene knockout or engineered drug target alleles (for example, *EML-ALK*) can be used for testing drug sensitivity or resistance in cultured cells or mice. **b** A lentiviral single guide RNA (sgRNA) library can be stably introduced into cells through pooled infection. Deep sequencing will measure sgRNA enrichment or depletion upon drug treatment to identify drug-resistant or drug-sensitive genes. **c** A diagram of genetic disease correction (adapted from [19]). A homology-directed repair (HDR) template single-stranded DNA carrying a wild-type ‘G’ nucleotide was applied to repair the ‘A’ mutation in the last position of exon 8 in the type I tyrosinemia gene *Fah*

Besides building various cancer models, the CRISPR-Cas9 system can also be used to explore drug treatment and resistance. In an *Arf*^*−/−*^*Eμ-myc* B-cell lymphoma mouse model, CRISPR-Cas9-mediated disruption of *p53* confers cells with resistance to doxorubicin treatment [42]. A new mouse model of Eml4-Alk-driven lung cancer engineered by CRISPR-mediated oncogenic chromosomal rearrangements has shown marked sensitivity to the Alk-inhibitor crizotinib [22]. These CRISPR mouse models provide a useful tool for exploring drug treatment and potential drug-sensitive or drug-resistant genes to advance therapeutic strategies for cancer patients (Fig. 4a).

Several groups have reported the successful implementation of the CRISPR-Cas9 system for large-scale high-throughput genetic screens. A pooled loss-of-function genetic screening approach has been described [50, 51]. In one study, a genome-scale lentiviral sgRNA library was used to generate knockout pools of cells and perform screens [51]. sgRNAs targeting a drug target gene essential for cell survival were depleted in the cell population. An sgRNA library targeting the *BCR* and *ABL1* genes was used to screen sgRNAs targeting the *BCR-ABL* fusion protein, which is the drug target of Gleevec, used to treat chronic myelogenous leukemia (CML) [51]. sgRNAs targeting the exons of the *BCR* and *ABL1* fusion genes were depleted in the screen, confirming that the BCR-ABL fusion protein is required for the survival of CML cells. This study suggests that CRISPR can be used to knockout candidate genes, accelerating the identification of novel drug target genes.

As mentioned above, CRISPR screens have been established to identify drug resistance genes on a genome-wide scale. For example, lentiviral delivery of a genome-wide CRISPR-Cas9 knockout library targeting 18,080 genes with 64,751 unique guide sequences enables both negative and positive selection screening in human cells [50]. This system has been used to screen for genes involved in resistance to vemurafenib, a therapeutic inhibitor of the oncogenic BRAF protein. The screening yielded high-ranking candidate genes that include previously validated genes such as *NF1* and *MED12* as well as novel hits *NF2*, *CUL3*,*TADA2B* and *TADA1* [50, 51]. Using the same CRISPR library screening method, two studies reported screens for resistance to the nucleotide analog 6-thioguanine and identified all the members of the DNA mismatch repair pathway, demonstrating that CRISPR genome-wide screening is a reliable approach to identify novel drug resistant genes and pathways (Fig. 4b) [51, 52].

Precise genome editing also holds great promise for correcting genetic disease genes [53]. We have shown that in vivo delivery of CRISPR components can correct the hereditary tyrosinemia gene *Fah* in the adult mouse liver [19] (Fig. 4c). Several groups have also demonstrated correction of genetic disease genes, such as the cystic fibrosis transmembrane conductance regulator (*CFTR*) gene in intestinal organoids [54], a dominant mutation in the *Crygc* gene that causes cataracts [55], and the Duchenne muscular dystrophy (*Dmd*) gene in mouse zygotes [56].

## Remaining challenges

Despite tremendous progress in the past 2 years with the CRISPR-Cas9 system and the generation of cancer models, a few challenges still remain. The major remaining obstacle is the delivery of Cas9 for building somatic mouse models and for using CRISPR-Cas9 to correct disease gene mutations in vivo. Improved CRISPR delivery (protein [57, 58] or mRNA) and split Cas9 [59, 60] will enhance the efficiency of in vivo genome editing. Finding alternative Cas9 proteins with smaller sizes [61–63] will also facilitate CRISPR viral vector packaging.

In addition, although CRISPR-Cas9 has been demonstrated as a flexible research tool, the safety of Cas9 remains a concern for the scientific community [64]. Future work is required to carefully evaluate the safety of CRISPR-Cas9.

Precision mouse models require minimal off-target effects. Several groups have developed technologies to reduce off-target genome editing, such as Cas9^D10A^ nickase and offset sgRNAs [65, 66], truncated sgRNA [67, 68] and dCas9-FokI fusions [69]. Genome-wide analysis of Cas9 off-target sites has been reported [70–72]. Monitoring off-target effects will be an important part of developing this method.

## Conclusions and future discussions

With the successful sequencing of human tumor genomes by large-scale efforts, such as TCGA, functional studies are required to conclusively identify novel bona fide tumor suppressor genes or oncogenes. The CRISPR system provides a fast, easy and reliable method to establish cancer models, explore drug treatments and identify various novel drug target genes and resistance genes [73]. As cancer involves complex alterations and mutations of tumor suppressor genes and oncogenes, CRISPR is a promising way to establish and investigate cancer models. At the same time, it will allow researchers to explore therapeutic strategies inspired by CRISPR-based genetic screens [74].

CRISPR has been used to establish different cancer mouse models using a variety of techniques. These include germline editing and somatic genome editing for modeling gene knockouts, knock-ins and chromosomal rearrangements. This has led to the creation of knockout and knock-in mouse models, solid tumor models and leukemia mouse models. The combination of CRISPR with traditional Cre-LoxP methods shows promise in the ongoing effort to establish precision mouse models to better understand the cooperative effects of tumor suppressor genes and oncogenes. CRISPR is also amenable for genome-wide screening to identify drug-resistant genes. CRISPR-mediated genome editing and genetic screens will facilitate cancer genomics and functional studies.

In the future, the CRISPR-Cas9 system will be more finely tuned, accelerating in vitro and in vivo genome editing to establish novel cancer models and better understand cooperative effects among complex tumor suppressor gene and oncogene networks. Furthermore, engineering new Cas9 fusion proteins with novel characteristics, such as light-inducible dCas9 [75, 76], could confer CRISPR with additional advantages. In vivo CRISPR screens [77] may identify new cancer driver genes in a similar fashion to in vivo RNA-interference-based approaches [78]. Apart from refining the CRISPR system, we may also be able to broaden the application of CRISPR to gene therapy, from fixing single-gene mutations to multiplex gene modifications to precisely edit the genome and eventually combat complex genetic diseases such as cancer and diabetes. This novel technology will continue to revolutionize modern biology. Furthermore, we envision the use of genome-editing approaches for generating cancer mouse models to understand the progression of individual tumors and to identify treatment strategies. Precision cancer mouse models will pave the road for precision cancer medicine.

## Box 1 Factors to consider when choosing a CRISPR approach

1. Predict top-ranking single guide RNAs (sgRNAs) using published tools such as CRISPR Design Tool [16], sgRNA Designer [79], E-CRISP [80], via the Cas-OFFinder [81] or CRISPRseek [82].

2. Clone sgRNA into pX330 vector [16] for transient expression or improved lentivectors [83] for stable expression.

3. Several groups have reported being able to increase genome-editing efficiency by microhomology-based choice of target sites [84,85] or by rational design of homology-directed repair (HDR) templates [86] and small molecular compounds [87,88].

4. A detailed protocol for generating a germline CRISPR mouse model was recently published [26].

5. For in vitro CRISPR, choose one sgRNA to introduce indels mediated by non-homology end joining (NHEJ) or precise point mutations mediated by HDR in the presence of template DNA in order to investigate a single gene knockout and establish genetically modified cell lines. For multiple gene modifications, multiple sgRNAs can be used to disrupt multiple genes.

6. Adenovirus, adeno-associated virus (AAV) and lentivirus can be used to deliver CRISPR in vivo to establish cancer mouse models [24], depending on the efficiency of infected tissues.

7. Two sgRNAs to generate two double-strand DNA breaks in the chromosomal regions of interest can be used to model large chromosomal deletions, inversions and translocations. A combination of Cre-LoxP and CRISPR can introduce additional genetic modification in available Cre-LoxP knockout or knock-in mouse models.

8. dCas9 without catalytic function fused with regulatory domains [89–94] and chimerical sgRNA scaffold [95,96] can be used in gene regulation studies.

9. Finally, CRISPR can be used for genome-wide screens to identify drug target genes or resistant genes using sgRNA libraries. This genome-wide screening strategy can also be adapted to identify novel tumor suppressor genes or oncogenes if properly designed.

## Abbreviations

AAV: adeno-associated virus
AML: acute myeloid leukemia
CML: chronic myelogenous leukemia
CRISPR: clustered regularly interspersed short palindromic repeats
dCas9: deactivated Cas9
Dox: doxycycline
DSB: DNA double-strand break
ES cell: embryonic stem cell
HDR: homology-directed repair
HSC: hematopoietic stem cell
HSPC: hematopoietic stem and progenitor cell
indel: insertion and deletion
kb: kilobase
NHEJ: non-homologous end joining
NIH: National Institutes of Health
NSCLC: non-small-cell lung cancer
PAM: protospacer-adjacent motif
sgRNA: single guide RNA
TALEN: transcription-activator-like effector nuclease
TCGA: The Cancer Genome Atlas
ZFN: zinc-finger nuclease
